# Ultrasound Features and Performance of Afirma Gene Sequencing Classifier in Cytologically Indeterminate Thyroid Nodules

**DOI:** 10.1210/jendso/bvae010

**Published:** 2024-01-31

**Authors:** Irina Azaryan, Mayumi Endo, Jennifer A Sipos, Jianing Ma, Jing Peng, Fadi Nabhan

**Affiliations:** Division of Endocrinology, Diabetes and Metabolism, The Ohio State University and Arthur G. James Cancer Center, Columbus, OH 43210, USA; Division of Endocrinology, Diabetes and Metabolism, The Ohio State University and Arthur G. James Cancer Center, Columbus, OH 43210, USA; Division of Metabolism, Endocrinology, and Nutrition, University of Washington, Seattle, WA 98105, USA; Division of Endocrinology, Diabetes and Metabolism, The Ohio State University and Arthur G. James Cancer Center, Columbus, OH 43210, USA; Center of Biostatistics and Bioinformatics, The Ohio State University, Columbus, OH 43210, USA; Center of Biostatistics and Bioinformatics, The Ohio State University, Columbus, OH 43210, USA; Division of Endocrinology, Diabetes and Metabolism, The Ohio State University and Arthur G. James Cancer Center, Columbus, OH 43210, USA

**Keywords:** thyroid nodules, molecular test, Afirma GSC

## Abstract

**Background:**

Cytologically indeterminate thyroid nodules (ITN) pose a management challenge. Here we analyze if adding ultrasound characteristics to Afirma Genome Sequence Classifier (GSC) results increases GSC diagnostic performance.

**Methods:**

We retrospectively analyzed 237 GSC-tested Bethesda III/IV ITNs between July 2017 and December 2019 and classified them by American Thyroid Association (ATA) and the Thyroid Imaging Reporting and Data System (TIRADS) of the American College of Radiology.

**Results:**

The benign call rate was higher in Bethesda III ITNs with TIRADS <5 vs TIRADS 5 (89% vs 68%. *P* = .015). The sensitivity, specificity, positive predictive value (PPV), and negative predictive value (NPV) of GSC in ATA high-risk Bethesda III ITNs vs lower were 100% vs 80% (*P* = 1), 89.5% vs 91.5% (*P* = .67), 66.7% vs 25% (*P* = .13), and 100% vs 99.2% (*P* = 1), respectively, and for TIRADS 5 vs <5 were 100% vs 80% (*P* = 1), 88.2% vs 91.4% (*P* = .65), 71.4% vs 23.5% (*P* = .06), and 100% vs 99.3% (*P* = 1). The sensitivity, specificity, PPV, and NPV of GSC in high-risk ATA Bethesda IV ITNs vs lower were 66.7% vs 100% (*P* = .42), 83.3% vs 85.7% (*P* = 1), 66.7% vs 64.3% (*P* = 1), and 83.3% vs 100% (*P* = .3), respectively, and for TIRADS 5 vs <5 were 66.7% vs 90% (*P* = .42), 88.9% vs 83.8% (*P* = 1), 66.7% vs 60% (*P* = 1), and 88.9% vs 96.9% (*P* = .39).

**Conclusion:**

Sensitivity, specificity, NPV, and PPV of GSC were not significantly different in ATA high-risk and TIRADS 5 ITNs compared to ATA < high-risk and TIRADS 1-4 ITNs.

Thyroid nodules are common, but only about 8% to 16% of thyroid nodules are cancerous [1]. Several sonographic classification systems have been used to simplify the reporting of thyroid nodules and to guide the need for fine needle aspiration (FNA) biopsy. The 2 most widely used classifications are that of the American Thyroid Association (ATA) and the Thyroid Imaging Reporting and Data System (TIRADS) of the American College of Radiology (ACR). The ATA 2015 classification of thyroid nodules is a pattern-based risk stratification system that relies on the constellation of features including nodule composition, shape, echogenicity, border, and presence of echogenic foci. Based on these patterns, the 2015 ATA system stratifies nodules into 5 categories, each with an associated risk of malignancy and, accordingly, different size thresholds for biopsy [2]. The ACR TIRADS assigns points for different ultrasound (US) features; the sum of these points determines the overall risk of malignancy and nodule size thresholds for biopsy [3].

Ultrasound-guided FNA is the gold-standard test for diagnostic assessment of thyroid nodules, yet cytologic evaluation may be limited with up to 30% of samples being classified as indeterminate using the widely accepted Bethesda classification [4]. Indeterminate thyroid nodules (ITN) are stratified into 3 categories in the Bethesda system: atypia or follicular lesion of undetermined significance (Bethesda III), follicular or Hurthle cell neoplasm (Bethesda IV), and suspicious for malignancy (Bethesda V). Nodules characterized as Bethesda III and Bethesda IV carry a malignancy risk of 6% to 18% and 10% to 40% respectively [5, 6]. Bethesda category V nodules have a 53% to 97% risk of malignancy and are usually treated with lobectomy or total thyroidectomy [7].

The diagnostic uncertainty associated with indeterminate thyroid cytology (Bethesda III/IV) presents a management challenge [4-7]. Traditionally options were limited to repeat biopsy or diagnostic thyroid lobectomy, though most of these nodules eventually prove to be benign on surgical pathology [8]. Several molecular tests (MTs) were developed to refine the preoperative diagnosis and reduce the rate of diagnostic surgeries [9, 10]. The Afirma Gene Sequencing Classifier (GSC) was developed to maintain the high sensitivity and negative predictive value (NPV) of the Gene Expression Classifier and improve upon its specificity and positive predictive value (PPV) [11, 12]. With a malignancy prevalence of 24% (Bethesda III/IV), the NPV for the GSC was 96% and PPV was 47%. High sensitivity was maintained for the GSC at 91% while the specificity improved from 48% to 68% for Bethesda III/IV samples [12].

In this study we aim to investigate whether combining US features and Afirma GSC results will better estimate the risk of malignancy in cytologically indeterminate nodules.

## Methods

This is a retrospective analysis of cytologically indeterminate thyroid nodules that underwent an Afirma GSC test from July 2017 to October 2019 at a single tertiary care academic medical center. The decision to perform FNA biopsy was made based on the clinical judgment of the physician and patient preference.

All FNAs were performed at The Ohio State University Medical Center using 23-, 25-, or 27- gauge needles under US guidance with samples collected for GSC analysis. Cytologic analysis was performed by pathologists at Ohio State. All specimens for molecular testing were stored in a −75° C freezer and were shipped to Veracyte, Inc. for GSC testing upon receipt of a Bethesda III or IV cytology result. It was generally recommended that patients with ITN and an GSC suspicious result undergo surgery and patients with GSC benign nodules observe with serial US examinations.

All thyroid US images were reviewed by 2 different endocrinologists with expertise in sonography. Each nodule was classified based on the ATA 2015 Sonographic Risk assessment and the ACR TIRADS 2017 classification system. Mutual consensus was obtained between the 2 reviewers in the event of discrepancy of the categorization, and agreement was reached upon discussion of the images. No nodules were excluded from the study due to discordant US assessments.

We categorized the nodules based on the GSC results: benign and suspicious. GSC suspicious nodules were further characterized by the surgical pathology results into malignant and benign histology. All GSC benign nodules with no surgical pathology available were considered truly benign. Nodules with histology of noninvasive follicular thyroid neoplasm with papillary like nuclear features (NIFTP) were considered malignant due to the current recommendations for management with surgery. Unoperated GSC suspicious nodules were excluded.

We also collected follow-up data of the GSC benign ITNs to determine the outcome of those nodules.

The study was approved by The Ohio State University Medical Center Institutional Board Review (IRB No 2017H0464).

### Statistical Analysis

GSC classification measures including sensitivity, specificity, PPV, and NPV were calculated for the overall sample and for Bethesda III and IV nodules separately with 95% Wilson confidence interval. The benign call rate (BCR) was calculated for the entire cohort and separately for Bethesda III and IV nodules. For statistical analysis, the sonographic classifications of nodules with ATA very low, low, and intermediate risk were combined into 1 group, called the ATA non-high-risk group. This group was compared to the ATA sonographically high-risk nodules. Similarly, the nodules with TIRADS scores of 1 to 4 were combined in 1 group, called the TIRADS <5. Nodules with a TIRADS score of 5 were analyzed as a separate group.

PPV and specificity of the GSC were calculated based on Bethesda classification and ATA and TIRADS classifications. Comparisons were performed using Chi-square test and Fisher's exact test between categorical variables. A *P*-value <.05 was deemed to be statistically significant.

## Results

**Figure 1.**
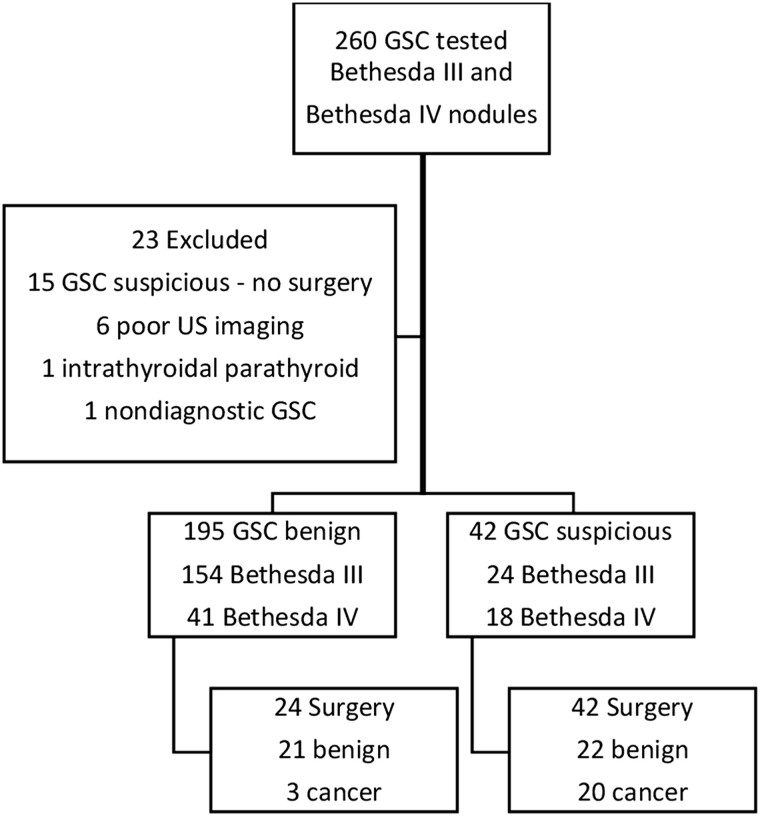
Study cohort. Follow-up data of GSC benign nodules are presented in Fig. 5. Abbreviations: GSC, Gene Sequencing Classifier.

The study cohort consisted of 260 thyroid nodules from 240 patients. Twenty-three nodules were excluded from the study: 15 GSC suspicious nodules that did not undergo surgery at the time of data collection, 6 nodules due to poor US images, 1 due to molecular result indicating parathyroid tissue, and 1 due to a nondiagnostic GSC result (Fig. 1). Our final cohort consisted of 237 cytologically indeterminate nodules (Bethesda III and IV) from 221 patients. Of those, 195 (82.3%) nodules had a GSC benign result and 42 (17.6%) had a GSC suspicious result.

Of the 42 GSC suspicious nodules, histopathology revealed cancer or NIFTP in 20 cases and benign pathology in 22 cases. Of the 195 nodules with Afirma GSC benign result, 24 (12.3%) were resected due to size increase on follow-up imaging. Histology was benign in 21 nodules and cancerous in 3 nodules (Fig. 1).

In the GSC benign group, 154 (79%) nodules had Bethesda III cytology (AUS/FLUS) and 41 (21%) nodules had Bethesda IV (false negative/suspicious for follicular neoplasm) cytology. In the GSC suspicious group, 24 (57%) nodules had Bethesda III cytology and 18 (43%) nodules had a Bethesda IV cytologic result (Fig. 1). There were 14 (5.9%) nodules that were nonclassifiable by the ATA US classification system. Of those, 11 had a GSC benign result and 3 were categorized as GSC suspicious. All nodules were scored using the TIRADS classification system. Patient and nodule characteristics for the entire cohort separated by GSC status are presented in Table 1. Patient and nodule characteristics for GSC suspicious subgroup as well as histopathology of malignant cases (n = 20) are summarized in Table 2.

**Table 1.** Patient/nodule characteristics by GSC status

	GSC benign nodules (n = 195)	GSC suspicious nodules (n = 42)
Age, median (IQR)	59 (46, 66)	46 (37, 59)
Sex, female (%)	150 (77)	32 (76)
Largest nodule dimension (cm), median (IQR)	2.1 (1.56, 2.73)	2.0 (1.7, 2.95)
Nodule volume (cm^3^), median (IQR)	2.1 (0.9, 4.9)	2.1 (1.2, 4.3)
ATA risk (%)		
Very low	22 (11)	1 (2.4)
Low	78 (40)	16 (38)
Intermediate	61 (31)	13 (31)
High	23 (12)	9 (21)
Unclassified	11 (5.6)	3 (7.1)
TIRADS (%)		
1	5 (2.6)	0 (0)
2	14 (7.2)	1 (2.4)
3	69 (35)	16 (38)
4	83 (43)	15 (36)
5	24 (12)	10 (24)
Bethesda III nodules (%)	154 (79)	24 (57)
AUS	104 (68)	16 (67)
FLUS	50 (32)	8 (33)
Bethesda IV nodules (%)	41 (21)	18 (43)
SFN	39 (95)	17 (94)
HCN	2 (4.9)	1 (5.6)

Abbreviations: ATA, American Thyroid Association; AUS, atypia of undetermined significance; FLUS, follicular lesion of undetermined significance; GSC, Gene Sequencing Classifier; HCN, Hurthle cell neoplasm; IQR, interquartile range; SFN, suspicious for follicular neoplasm; TIRADS, Thyroid Imaging Reporting and Data System.

Median (IQR): n (%).

**Table 2.** GSC suspicious (n = 42) nodules characteristics by pathology status

	Malignant pathology (n = 20)	Benign pathology (n = 22)
Age, median (IQR)	50 (39, 60)	44 (37, 57)
Sex, female (%)	17 (85)	15 (68)
Largest nodule dimension (cm), median (IQR)	2.05 (1.34, 3.03)	2.0 (1.7, 2.5)
Nodule volume (cm^3^), median (IQR)	2.0 (1, 5)	2.0 (2, 4)
ATA risk (%)		
Very low	0 (0)	1 (4.5)
Low	6 (30)	10 (45)
Intermediate	7 (35)	6 (27)
High	6 (30)	3 (14)
Unclassified	1 (5)	2 (9)
TIRADS (%)		
2	0 (0)	1 (4.5)
3	6 (30)	10 (45)
4	7 (35)	8 (36)
5	7 (35)	3 (14)
Bethesda III nodules (%)	9 (45)	15 (68)
AUS	7 (78)	9 (60)
FLUS	2 (22)	6 (40)
Bethesda IV nodules (%)	11 (55)	7 (32)
SFN	10 (91)	7 (100)
HCN	1 (9)	0 (0)
Histopathology	Classic variant PTC: 8 Follicular variant PTC: 6 Oncocytic variant PTC: 1 FTC: 3 NIFTP: 2	

Abbreviations: ATA, American Thyroid Association; AUS, atypia of undetermined significance; FLUS, follicular lesion of undetermined significance; FTC, follicular thyroid carcinoma; GSC, Gene Sequencing Classifier; HCN, Hurthle cell neoplasm; IQR, interquartile range; NIFTP, noninvasive follicular thyroid neoplasm with papillary like nuclear features; PTC, papillary thyroid carcinoma; SFN, suspicious for follicular neoplasm; TIRADS, Thyroid Imaging Reporting and Data System.

Median (IQR); n (%).

**Figure 2.**
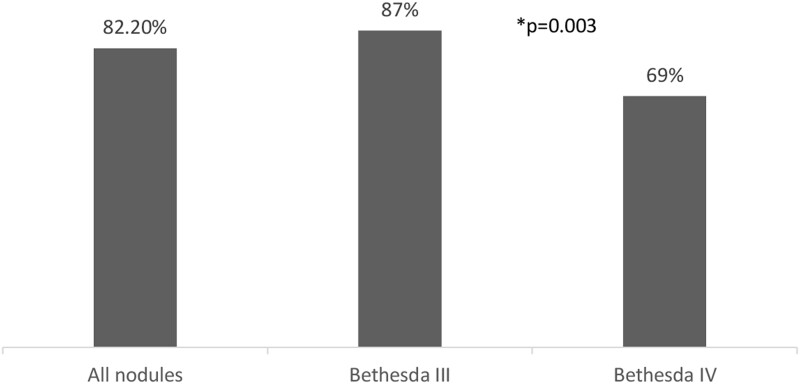
Benign call rate for all nodules and for Bethesda III and Bethesda IV groups. *P < .05.

**Figure 3.**
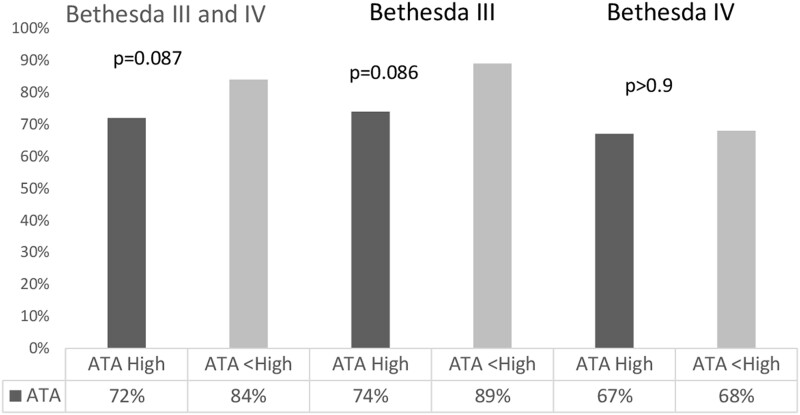
Benign call rate for the nodules classified per ATA. *P < .05. Abbreviations: ATA, American Thyroid Association.

The BCR of the GSC test (the rate at which GSC was read as benign) in all ITNs was 82.2% (195/237). The BCR was significantly higher in Bethesda III nodules at 87% compared to Bethesda IV nodules at 69% (*P* = .003) (Fig. 2). To understand if the BCR varies according to the sonographic (US) appearance of ITNs, we analyzed the BCR in ITNs that were considered high risk based on ATA classification compared to ATA non-high-risk nodules (ATA unclassified nodules were excluded from this analysis, n = 14). In the entire cohort, the BCR was higher in nodules in the ATA non-high-risk group than in the ATA high-risk group (84% vs 72%, *P* = .087). Similarly, in nodules with Bethesda III cytology, the BCR was higher for ATA non-high-risk US nodules compared to the ATA high-risk nodules (89% vs 74%, *P* = .086), though this difference did not reach statistical significance. In nodules with Bethesda IV cytology, there was no difference in the BCR for the ATA non-high-risk group compared to the ATA high-risk group (68% vs 67%. *P* > .9) (Fig. 3).

**Figure 4.**
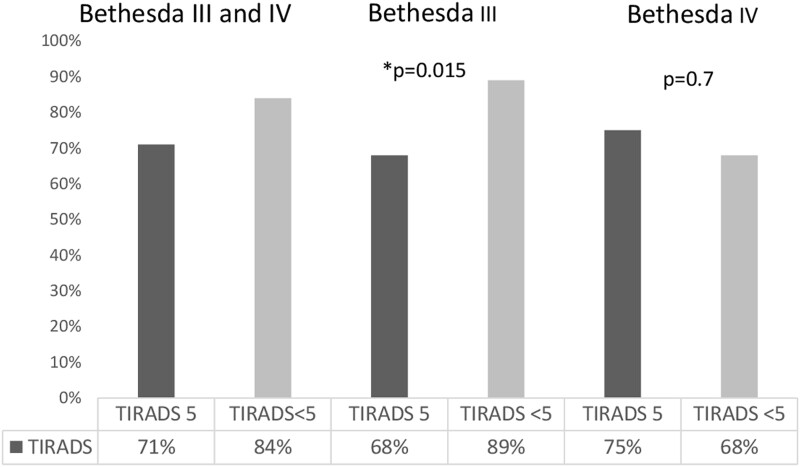
Benign call rate for the nodules classified per TIRADS. *P < .05. Abbreviations: TIRADS, Thyroid Imaging Reporting and Data System.

We then applied the same analysis using the ACR-TIRADS US features. The BCR was higher for TIRADS <5 nodules compared to TIRADS 5 nodules (84% vs 71% *P* = .054). Among nodules with Bethesda III cytology, the BCR was significantly higher in the TIRADS <5 group compared to TIRADS 5 nodules (89% vs 68%. *P* = .015). Among nodules with Bethesda IV cytology, the BCR was not significantly different in the TIRADS <5 group compared to TIRADS 5 nodules (68% vs 75% *P* = .7) (Fig. 4).

### Sensitivity, Specificity, PPV, and NPV of GSC Test

Assuming all unoperated GSC benign cases were truly benign, the sensitivity, specificity, PPV, and NPV of the GSC test for all nodules were 87%, 89.7%, 47.6%, and 98.5%, respectively, with a disease prevalence of 9.7% (Table 3). The sensitivity, specificity, PPV, and NPV of the GSC test for Bethesda III and Bethesda IV nodules are presented in Table 3. To understand if the GSC performance varies according to the US appearance of ITNs, we analyzed the sensitivity, specificity, PPV, and NPV for nodules with ATA high-risk patterns compared to nodules with ATA non-high-risk US patterns (ATA unclassified nodules were excluded from this analysis, n = 14). The diagnostic performance of the GSC test in ATA high-risk compared to ATA non-high-risk nodules for all ITNs and for Bethesda III and IV nodules did not show statistically significant difference (Table 4). We then applied the same analysis using the ACR-TIRADS US features instead of ATA. The sensitivity, specificity, PPV, and NPV of the GSC test in nodules that had a TIRADS 5 score compared to nodules with a <5 TIRADS score for all ITNs and for Bethesda III and Bethesda IV nodules are presented in Table 5. The PPV of the GSC test in Bethesda III nodules with a TIRADS 5 score compared to <5 TIRADS was close to statistically significant difference: 71.4% vs 23.5% (*P* = .06). No significant difference was reported in other classification measures of the GSC test in all ITNs or Bethesda III and IV nodules after applying ACR-TIRADS US features (Table 5).

**Table 3.** Classification measures of GSC test in the entire cohort

Bethesda classification	Sensitivity, % (95% CI)	Specificity, % (95%CI)	PPV, % (95%CI)	NPV, % (95%CI)	TP	FP	TN	FN	n
III and IV	87 (66.4-97.2)	89.7 (84.8-93.4)	47.6 (32 -63.6)	98.5 (95.6-99.7)	20	22	192	3	237
III only	90 (55.5 -99.7)	91.1 (85.7-94.9)	37.5 (18.8-59.4)	99.4 (96.4-100)	9	15	153	1	178
IV only	84.6 (45.6 -98.1)	85.4 (71.1-93.7)	61.1 (35.7-82.7)	95.1 (83.5-99.4)	11	7	39	2	59

Abbreviations: CI, confidence interval; FN, false negative; FP, false positive; GSC, Gene Sequencing Classifier; n, total nodules; NPV, negative predictive value; PPV, positive predictive value; TN, true negative; TP, true positive.

**Table 4.** Classification measures of the GSC test per ATA ultrasound features

ATA	Sensitivity, % (95% CI)	Specificity, % (95%CI)	PPV, % (95% CI)	NPV, % (95% CI)	TP	FP	TN	FN	n
Bethesda III and IV nodules									
ATA High	85.7 (42.1-99.6)	88 (68.8-97.5)	66.7 (29.9-92.5)	95.7 (78.1-99.9)	6	3	22	1	32
ATA <High	86.7 (59.5-98.3)	90.3 (85-94.3)	43.3 (25.5-62.6)	98.8 (95.6-99.8)	13	17	159	2	191
Bethesda III nodules									
ATA High	100 (39.8-100)	89.5 (66.9-8.7)	66.7 (22.3-95.7)	100 (80.5-100)	4	2	17	0	23
ATA <High	80 (28.4-99.5)	91.5 (85.7-95.6)	25 (7.3-52.4)	99.2 (95.8-100)	4	12	130	1	147
Bethesda IV nodules									
ATA High	66.7 (9.4-99.2)	83.3 (35.9-99.6)	66.7 (29.9-92.5)	83.3 (35.9 -99.6)	2	1	5	1	9
ATA <High	100 (66.4-100)	85.7 (69.7-95.2)	64.3 (35.1-87.2)	100 (88.4-100)	9	5	29	1	44

Abbreviations: ATA, American Thyroid Association; CI, confidence interval; FN, false negative; FP, false positive; GSC, Gene Sequencing Classifier; n, total nodules; NPV, negative predictive value; PPV, positive predictive value; TN, true negative; TP, true positive.

*P < .05.

No statistical difference was reported in sensitivity, specificity, PPV, and NPV between different groups.

**Table 5.** Classification measures of the GSC test per TIRADS ultrasound categorization

ATA	Sensitivity, % (95% CI)	Specificity, % (95% CI)	PPV, % (95% CI)	NPV, % (95% CI)	TP	FP	TN	FN	n
Bethesda III and IV nodules									
ATA High	85.7 (42.1-99.6)	88 (68.8-97.5)	66.7 (29.9-92.5)	95.7 (78.1-99.9)	6	3	22	1	32
ATA < High	86.7 (59.5-98.3)	90.3 (85-94.3)	43.3 (25.5-62.6)	98.8 (95.6-99.8)	13	17	159	2	191
Bethesda III nodules									
ATA High	100 (39.8-100)	89.5 (66.9-98.7)	66.7 (22.3-95.7)	100 (80.5-100)	4	2	17	0	23
ATA < High	80 (28.4-99.5)	91.5 (85.7-95.6)	25 (7.3-52.4)	99.2 (95.8-100)	4	12	130	1	147
Bethesda IV nodules									
ATA High	66.7 (9.4-99.2)	83.3 (35.9-99.6)	66.7 (29.9-92.5)	83.3 (35.9-99.6)	2	1	5	1	9
ATA < High	100 (66.4-100)	85.7 (69.7-95.2)	64.3 (35.1-87.2)	100 (88.4-100)	9	5	29	1	44

Abbreviations: ATA, American Thyroid Association; CI, confidence interval; FN, false negative; FP, false positive; GSC, Gene Sequencing Classifier; n, total nodules; NPV, negative predictive value; PPV, positive predictive value; TIRADS, Thyroid Imaging Reporting and Data System; TN, true negative; TP, true positive.

*P < .05.

No statistical difference was reported in sensitivity, specificity, PPV, and NPV between different groups.

### Follow up of GSC Benign Nodules

**Figure 5.**
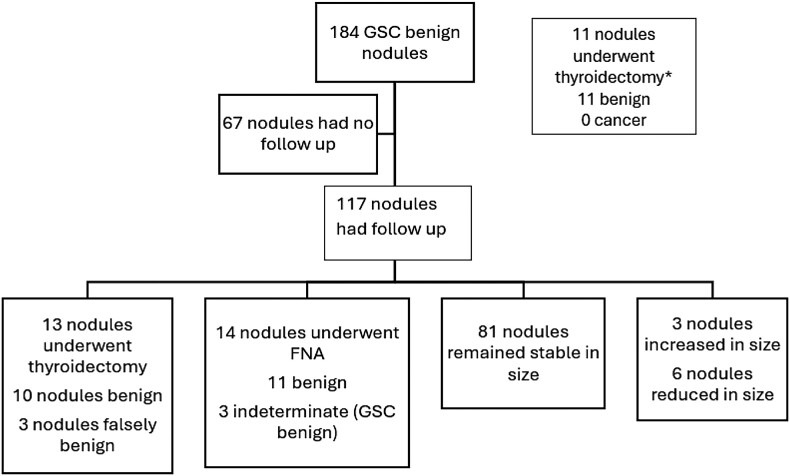
Follow-up of GSC benign nodules. This figure shows follow-up of GSC benign nodules with the median follow-up period of 34 months (IQR 18, 42). *Eleven out of 195 nodules underwent resection with the median of 4 months (IQR 2, 6) after the biopsy, leaving 184 nodules for long-term follow-up. Abbreviations: GSC, Gene Sequencing Classifier; IQR, interquartile range.

Eleven of the 195 GSC benign nodules underwent surgery within a median time of 4 months (interquartile range 2, 8) after the biopsy. Of the remaining 184 GSC benign nodules, 67 (36%) nodules were lost to follow-up and had no repeat thyroid imaging. One hundred seventeen (63%) GSC benign nodules had a follow-up with a median period of 34 months (interquartile range 18, 42). Of those nodules, 30 (25%) demonstrated an increase in size, 6 (5%) nodules became smaller in size, and 81 (69%) nodules remained stable during US surveillance. Of the 30 enlarging nodules, 13 underwent thyroidectomy; the histopathology of 10 nodules were benign, 1 nodule showed NIFTP, 1 nodule was classic papillary thyroid carcinoma, and 1 nodule was follicular thyroid carcinoma. Fourteen of the enlarging nodules underwent a repeat FNA biopsy with cytology results showing a benign (n = 11) or indeterminate result (n = 3) followed by a benign GSC result. The remaining 3 nodules with a size increase were followed with US surveillance (Fig. 5).

## Discussion

In our study we aimed to investigate whether adding ATA and TIRADs US classification of the nodule to the Afirma GSC result improves the diagnostic performance of the GSC in ITNs.

The BCR in our cohort of Bethesda III nodules was significantly higher than in Bethesda IV nodules (87% vs 69%, *P* = .003). In all ITNs, the BCR was higher in ATA non-high-risk nodules compared to those with ATA high-risk US features (84% vs 72%, *P* = .087) and TIRADS 1-4 nodules compared to those with a TIRADS 5 score (84% vs 71%, *P* = .054). A significant difference in BCR was noted among nodules with Bethesda III cytology and TIRADS 1-4 score compared to those with a 5 TIRADS score (89% vs 68%, *P* = .015). We did not see a significant difference in BCR of ITNs with Bethesda IV cytology. This finding may be helpful in either the selection of which nodule to send for molecular testing or to inform patients of the likelihood that a MT would change the management.

Several studies have demonstrated that US features are useful in estimating the risk of malignancy in thyroid nodules with indeterminate cytology [13-17]. In a retrospective study of 463 Bethesda III and IV nodules reported by Valderrabano et al, the odds ratio for malignancy was 5.18 (*P* < .001) in ATA high suspicion nodules compared to low/intermediate-risk nodules [13]. In another study by Ahmadi et al, the risk of malignancy in ATA high suspicion and TIRADS 5 nodules was 100% in Bethesda III nodules and 66.7% and 50% among Bethesda IV nodules for ATA high suspicion and TIRADS 5 nodules, respectively [14]. However, there are only few studies investigating whether the combination of the MT result and US findings would better predict malignancy risk in cytologically indeterminate thyroid nodules [18, 19].

In a prospective study evaluating 375 indeterminate thyroid nodules, the malignancy rates in ATA low/intermediate suspicion nodules increased from 21.0% to 56.3% (*P* < .0001) with a suspicious Afirma GSC result. However, the malignancy rate in ATA high suspicion nodules was not significantly increased by a suspicious Afirma GSC result: 77.8% to 87.5% (*P* = 1.0) [18]. In a recently published multicenter study of 257 nodules with indeterminate cytology and ThyroSeq V3 (TSv3) genomic classifier, the ATA (*P* = .1211) and TIRADS (*P* = .1359) US classifications did not show statistically significantly increased risk of cancer/NIFTP beyond that predicted by TSv3 [19]. These results are consistent with those of our analysis whereby we did not observe a statistically significant difference in PPV or specificity for the entire cohort or among Bethesda III and IV nodules after applying ATA and TIRADS US classification. We did, however, demonstrate a tendency toward a significance with a higher PPV in nodules with Bethesda III cytology and TIRADS 5 scores compared to the TIRADS 1-4 group, 71.4% vs 23.5% (*P* = .06). Further study with a larger cohort may provide additional statistical strength to support a relationship between the sonographic appearance and the GSC result for nodules with Bethesda III cytology.

There are several limitations to our study. Due to the retrospective design, there is potential for sample bias. The prevalence of cancer in our cohort is lower than the expected range among Bethesda III and IV nodules. This was likely caused by the fact that Bethesda III and IV nodules with clinically or sonographically suspicious features had a higher possibility of undergoing surgery without MT and therefore would have been excluded from our study. Other limitations include the relatively small size of the GSC suspicious surgical cohort, which may have impacted the significance of association.

Our study has important strengths. All US images were independently reviewed and classified by 2 different endocrinologists with significant experience in sonographic evaluation of thyroid nodules. GSC suspicious unresected nodules were excluded from the final analysis, and surgical histopathology was available in all included GSC suspicious nodules. Additionally, 63% percent of GSC benign nodules had a follow-up with a median period of 34 months.

In conclusion, in our cohort we observed a statistical difference in the BCR for Bethesda III cytology with a higher PPV in ATA high-risk and TIRADS 5 nodules. We did not observe a statistical change in PPV or specificity of GSC test based on the ATA or TIRADS US classification system. Additional studies with a larger cohort of GSC suspicious surgical cases may be required to determine if a more selective approach to MT can be applied based on US classification.

## Data Availability

All datasets generated and analyzed during the current study are available from the corresponding author from a reasonable request.
