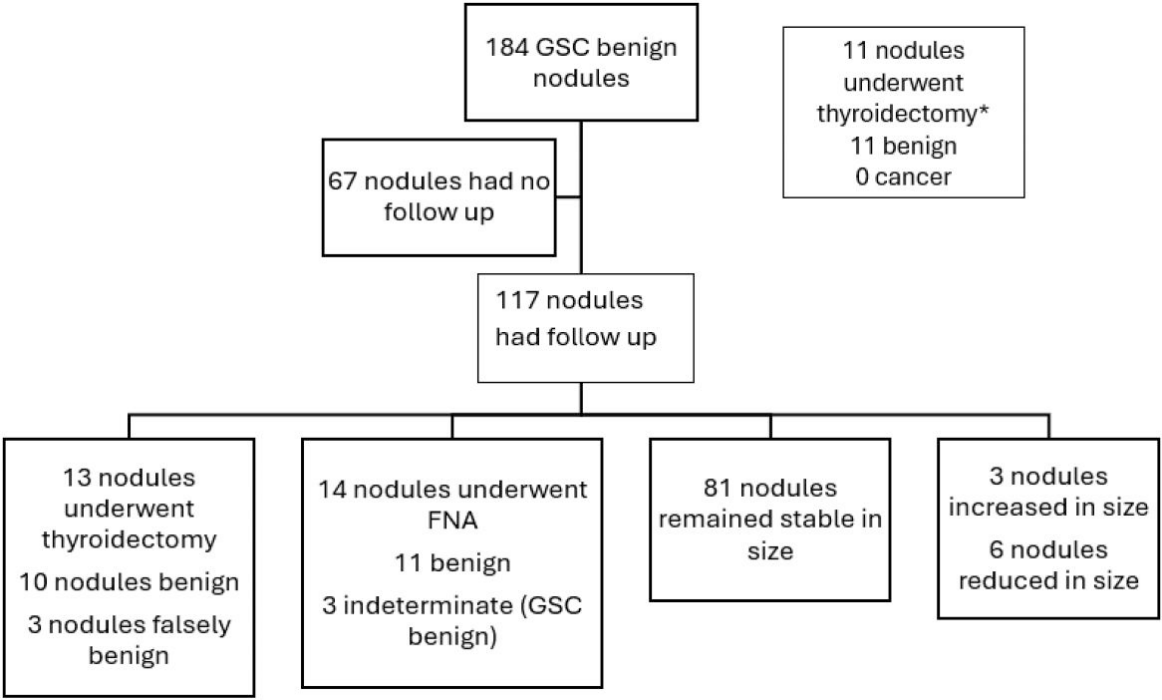# Correction to: “Ultrasound Features and Performance of Afirma Gene Sequencing Classifier in Cytologically Indeterminate Thyroid Nodules”

**DOI:** 10.1210/jendso/bvae216

**Published:** 2024-12-18

In the above-named article by Azaryan I, Endo M, Sipos JA, Ma J, Peng J, and Nabhan F (*J Endo Society*. 2024; 8(3); doi: 10.1210/jendso/bvae010), there were errors in figure 5.

In the originally published article, the flow diagram in Figure 5 accidentally represented 11 nodules that underwent thyroidectomy as a subset of 117 nodules that had follow up. The authors have provided a replacement for Figure 5 to clarify that the 11 nodules that underwent thyroidectomy were separate from the 117 that had follow up. The new Figure 5 also corrects two misspellings in the originally published version: “bening” has been corrected to “benign,” and “thyroidectoy” has been corrected to “thyroidectomy.”

The article has been corrected online.

doi: 10.1210/jendso/bvae010

Original Figure 5:

**Figure:**
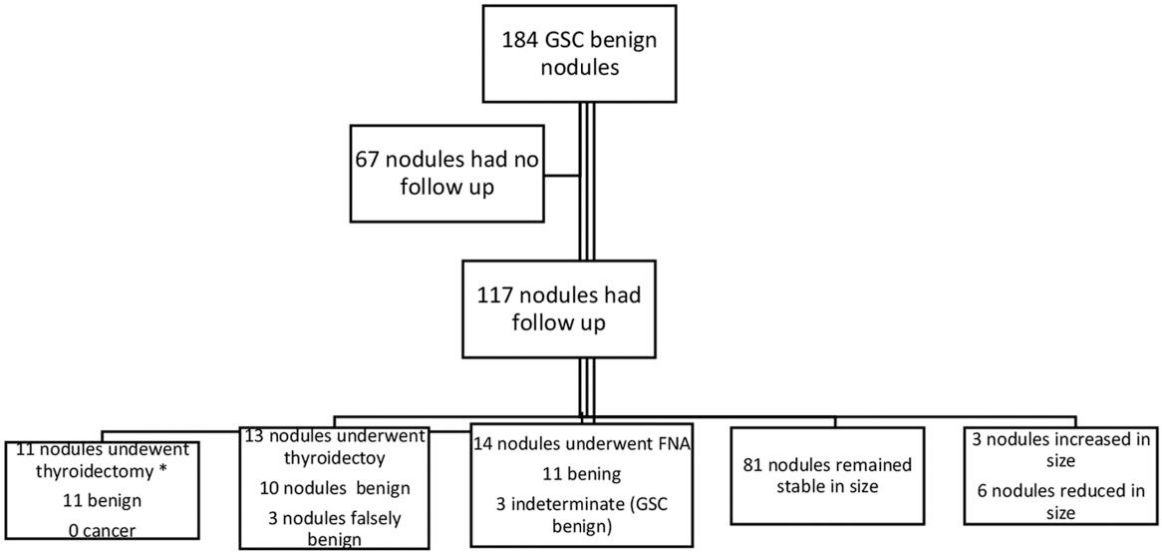


Corrected Figure 5:

**Figure:**